# L-Malate's Plasma and Excretion Profile in the Treatment of Moderate and Severe Hemorrhagic Shock in Rats

**DOI:** 10.1155/2016/5237148

**Published:** 2016-06-15

**Authors:** Indra N. Waack, Stephan Himmen, Friederike Mueller, Ricarda Rohrig, Friederike Roehrborn, Johanna K. Teloh, Herbert de Groot

**Affiliations:** Institute of Physiological Chemistry, University Hospital Essen, University of Duisburg-Essen, Hufelandstraße 55, 45147 Essen, Germany

## Abstract

*Introduction*. Malate is a standard component in fluid therapy within a wide range of medical applications. To date, there are insufficient data regarding its plasma distribution, renal excretion, and metabolism after infusion. This study aimed to investigate these three aspects in a rat model of moderate and severe hemorrhagic shock (HS).* Methods*. Male Wistar rats were subjected to HS by dropping the mean arterial blood pressure to 25–30 mmHg (severe) and 40–45 mmHg (moderate), respectively, for 60 minutes. Subsequently, reperfusion with Ringer-saline or a malate containing crystalloid solution (7 mM, 13.6 mM, and 21 mM, resp.) was performed within 30 minutes, followed by an observation period of 150 minutes.* Results*. In the present experiments, malate rapidly disappeared from the blood, while only 5% of the infused malate was renally excreted. In the resuscitation interval the urinary citrate and succinate amounts significantly increased compared to control.* Conclusion*. Malate's half-life is between 30 and 60 minutes in both, moderate and severe HS. Thus, even under traumatic conditions malate seems to be subjected to rapid metabolism with participation of the kidneys.

## 1. Introduction

Malate, a citric acid cycle (TCA) intermediate, is a standard component in fluid therapy in a wide spectrum of medical applications. In human medicine it is used as an integrant in a concentrate to treat hypokalemia (e.g., Kalium-L-Malat 17.21%, B. Braun Melsungen), in amino acid solutions for parenteral nutrition (e.g., Aminosteril® plus, Fresenius Kabi), in solutions, which are used to cover water, electrolyte, and energy requirements (e.g., Jonosteril® Na100 with glucose, Fresenius Kabi), and even in pediatrics (e.g., Paediatrische Elektrolytloesung 1, Fresenius Kabi). Furthermore, malate is the central component in Jonosteril Malat (Fresenius Kabi) that is appropriate to serve as primary volume therapy in emergency medicine and fluid replacement in cases of moderate acidosis and for the maintenance of pre-, intra-, and postoperative fluid balance. In the guidelines of the German Society of Trauma, the application of crystalloid solutions like Ringer-lactate, Ringer-acetate, and Ringer-malate is recommended [1], with respect to the field of emergency medicine, to treat hemorrhage, caused by accidental trauma, and the consecutively following life-threatening consequences which represent one of the main reasons of preventable death [2–4]. In addition, in the guidelines of the German Society of Anaesthesiology and Intensive Care Medicine balanced solutions containing malate or acetate are recommended for the treatment of critically ill patients, whereas lactate is not [5]. Malate's positive effects in certain clinical approaches, including investigations as regards its effect on energy metabolism, cardioprotection, fibromyalgia, physical stamina, and cancer, have been shown [6–13]. Even though research regarding the treatment of hemorrhagic shock (HS) and its consequences is very scarce, the protection by administering malate is described as well [14–16]. In contrast, treatment of severe HS with Ringer-lactate is debatable, since earlier studies showed detrimental effects in a rat model of severe HS [17, 18], whereas acetate-based balanced salt solution appeared to be predominant [19]. Certainly, acetate is reported to have vasodilatory effects resulting in cardiovascular depression* in vitro* as well as* in vivo* [20–22]. On the other hand, it is postulated that malate's metabolism is decelerated compared to that of acetate [23, 24], but not confirmed in hemorrhagic shock.

Though the previous examinations cited above yielded encouraging results to date and malate being a standard component of intravenous fluid therapy in a wide range of medical applications, information about malate's plasma distribution and excretion profile and its metabolism after incorporation into the tissues is very scarce. Therefore, the objective of the present study was to investigate malate's plasma distribution as well as renal excretion and its metabolic fate in the experimental models of severe and moderate HS. HS was chosen because of malate's potential to protect against shock-induced damage [15], thereby becoming relevant in the preclinical and early hospital therapy of traumatic bleeding.

## 2. Materials and Methods

### 2.1. Animals

Altogether, 48 male Wistar rats (420 g–500 g, age 12–14 weeks) were obtained from the central animal unit of the Essen University Hospital. Animals were kept under standardized conditions of temperature (22°C ± 1°C), humidity (55%  ±  5%), and 12/12-hour light/dark cycles. They were fed* ad libitum *(ssniff-Spezialdiaeten, Soest, Germany) with free access to water and not fasted before the experiments.

Experiments were conducted in accordance with the standards of Annex III of the directive 2010/63/EU of the European Parliament and of the Council of 22 September 2010 on the protection of animals used for scientific purposes [25]. The experimental protocol was reviewed and approved by the local Animal Care and Use Committee (Animal Care Center, University of Duisburg-Essen, Essen, Germany, and the district government of Duesseldorf (“North Rhine-Westphalia State Environment Agency”, Recklinghausen, Germany)) with a Permit Number 84-02.04.2012.A341, G1318/12.

### 2.2. Chemicals/Materials

Saline solution (NaCl, 0.9%) was provided by B. Braun (Melsungen, Germany), Ringer-malate (MR, Jonosteril Malat, 13.6 mM L-Malic acid) and Ringer-saline (RS) were from Fresenius (Bad Homburg, Germany), and acid-citrate-dextrose A solution (ACD-A) was from Fenwal (Lake Zurich, IL, USA). NaCl and KCl were provided by Carl Roth (Karlsruhe, Germany), CaCl_2_-dihydrate, MgCl_2_-hexahydrate, and NaOH were provided from Merck (Darmstadt, Germany), and L(−)-malic acid monosodium salt (Na-Mal) was from ApplyChem (Darmstadt, Germany). Isoflurane (Forene) was obtained from Abbott (Wiesbaden, Germany), ketamine 10% from Ceva (Duesseldorf, Germany), and lidocaine (Xylocain 1%) from AstraZeneca (Wedel, Germany). Medical oxygen was from Air Liquide (Duesseldorf, Germany). Portex catheters (inner diameter: 0.58 mm, outer diameter: 0.96 mm) were provided from Smiths Medical International (Hythe, UK), Minisart was from sartorius stedim biotech (Goettingen, Germany), and Vasofix Safety (22G, 0.9 × 25 mm) and peripheral venous catheters were from B. Braun.

Since the higher (21 mM) and lower (7 mM) MR solutions were not commercially available, they had to be self-produced (7 mM: 119.78 mM NaCl, 5.4 mM KCl, 0.91 mM CaCl_2_-dihydrate, 1 mM MgCl_2_-hexahydrate, 7 mM NaOH and 21 mM: 97.54 mM NaCl, 5.4 mM KCl, 0.91 mM CaCl_2_-dihydrate, 1 mM MgCl_2_-hexahydrate, and 21 mM NaOH, resp.). After solving, the produced solutions were filtrated through sterile filters (Minisart, hydrophilic syringe filter, pore size: 0.2 *µ*m) into sterilized glass bottles (Schott, Mainz, Germany) and stored at 4°C for later use (storage maximum: 7 days).

### 2.3. Anesthesia, Analgesia, and Surgical Procedures

Anesthesia, analgesia, catheter insertion, and blood sampling were performed as described previously [19]. The rats were initially anesthetized with isoflurane (2.0% in 100% medical O_2_ at 4.0 L/min) in an induction chamber. Throughout the experiment animals were kept anesthetized through a face mask (1.0–2.0% isoflurane in 100% medical O_2_ at 1.0 L/min) connected to a vaporizer (Isoflurane Vet. Med. Vapor, Draeger, Luebeck, Germany) and received ketamine (50 mg/kg body weight subcutaneously) into the right chest wall for analgesia. After local lidocaine administration (5 mg/kg body weight subcutaneously), a skin-deep inguinal incision of about 2-3 cm was made, femoral vessels were freed from tissue (sparing the femoral nerve), and a Portex catheter was placed within the right femoral artery and the right femoral vein. Again after local lidocaine administration (5 mg/kg body weight subcutaneously) a median laparotomy of about 2 cm was performed to catheterize the bladder to obtain urinary samples. For the period of resuscitation, the prolonged infusion of 0.9% NaCl solution (5 mL/kg body weight × h, 37°C), to compensate intraoperative fluid depletion over surgical areas and the respiratory epithelium, was interrupted. At the end of the experiment, animals were sacrificed by resection of the heart under deep isoflurane anesthesia (4.0% isoflurane in 100% medical O_2_ at 1.0 L/min).

### 2.4. Induction of Hemorrhagic Shock and Resuscitation Process

The induction of hemorrhagic shock and resuscitation regimens were performed as previously described [18]. Briefly, hemorrhagic shock was induced at *T* = 30 min by removing either 2 mL (severe shock) or 1 mL (moderate shock) blood every three minutes through the femoral artery catheter using a 2 mL syringe (Becton, Dickinson and Company, Franklin Lakes, NJ, USA). Blood from the first withdrawal was used for assessment of blood and plasma parameters via blood gas analysis. The second syringe was prefilled with either 0.2 mL (severe shock) or 0.1 mL (moderate shock) of ACD-A solution. The syringe with citrated blood was stored at 37°C and used to regulate shock severity, if needed. Blood withdrawal was continued until the mean arterial blood pressure (MAP) dropped to (1) 25–30 mmHg (severe shock) or (2) 40–45 mmHg (moderate shock); shock induction typically took about 20 min. The animals in the comparative groups all possessed a similar weight (420 to 500 g), resulting in a nearly identical volume of the shed blood. The blood volume withdrawn was about (1) 13.9 mL (severe shock) or (2) 11.5 mL (moderate shock). For the next 60 min, the MAP was retained, usually without further intervention. In some cases, small amounts (0.1–0.5 mL aliquots) of the citrated blood had to be administered, or additional small blood samples (0.5–1.0 mL aliquots) had to be withdrawn, to keep the MAP within the desired range. After the shock phase, study group-specific resuscitation fluids were infused into the femoral vein within 30 min using a syringe pump (Perfusor Secura FT, B. Braun) in a randomized and blinded manner. Experiments were continued for another 150 min or until the rat died.

### 2.5. Experimental Groups

The following experimental groups were compared:

- Severe shock/RS (shock, resuscitation with RS, six animals).
- Severe shock/MR7 (shock, resuscitation with 7 mM MR, six animals).
- Severe shock/MR13.6 (shock, resuscitation with 13.6 mM MR, six animals).
- Severe shock/MR21 (shock, resuscitation with 21 mM MR, six animals).
- Moderate shock/RS (shock, resuscitation with RS, six animals).
- Moderate shock/MR7 (shock, resuscitation with 7 mM MR, six animals).
- Moderate shock/MR13.6 (shock, resuscitation with 13.6 mM MR, six animals).
- Moderate shock/MR21 (shock, resuscitation with 21 mM MR, six animals).

The composition of the two Ringer-based solutions with the higher and lower malate concentrations (MR7, MR21) are listed in Chemicals/Materials. The volume of the resuscitation fluids is based on the 3 : 1 rule (3 times the volume of the shed blood volume) [26].

### 2.6. Biomonitoring

Systolic blood pressure, diastolic blood pressure, and MAP were displayed on a monitor and documented continuously, by using the femoral artery catheter, which was connected to a pressure transducer. To keep the catheter functional RS was infused at a rate of 3 mL/h. Heart rate was determined from systolic blood pressure spikes. Recording of MAP and heart rate was intended to monitor an adequate anesthesia and to supervise the particular blood pressure (see above). These parameters were shown elsewhere [18]. The core body temperature of all rats was monitored using a rectal sensor and was maintained around 37.3°C to 37.6°C during the whole experiment via an underlying thermostat-controlled operating table and by covering the animal with aluminum foil. Peripheral oxygen saturation was recorded continuously using a pulse oximeter (OxiCliq A, Nellcor, Boulder, CO, USA) placed at the left hind limb. All parameters were recorded periodically every 10 min (exception at *T* = 125, 5 minutes in resuscitation).

### 2.7. Assessment of Blood and Plasma Parameters

Using a 2 mL syringe containing 80 IU electrolyte-balanced heparin, blood samples (0.5 mL, except of shock induction: 2 mL) were taken from the femoral artery at

(i) *T* = 0 min (immediately after insertion of the arterial catheter),
(ii) *T* = 30 min (start of shock induction),
(iii) *T* = 60 min (end of shock induction),
(iv) *T* = 120 min (start of resuscitation),
(v) *T* = 150 min (end of resuscitation),
(vi) *T* = 180, 240, and 300 min (observation phase).

For each blood sampling the animals were substituted with a 0.5 mL bolus of 0.9% NaCl solution via the femoral artery compensating the lost volume and keeping the catheter functional. In order to obtain plasma for the assessment of marker enzyme activities, blood was centrifuged at 4000 ×g for 15 min at room temperature. The gained plasma was aliquoted (50 *µ*L) and stored at −80°C until further examination. The plasma activity of lactate dehydrogenase (LDH) as a general marker for cell injury, creatine kinase (CK) activity as a marker for muscle cell injury, aspartate transaminase (ASAT) and alanine transaminase (ALAT) activities as marker for liver injury, and creatinine and urea as markers for renal injury were determined with a fully automated clinical chemistry analyzer (respons920, DiaSysDiagnostic Systems GmbH, Holzheim, Germany).

### 2.8. Assessment of Urine Parameters

Urinary samples were collected during five fixed time intervals, starting with the interval *T* = 0–30 min (immediately after catheter insertion and before shock induction). The subsequent intervals were the shock induction phase (*T* = 30–60 min), resuscitation phase (*T* = 120–150 min), and two intervals during the observation phase (*T* = 180–240 min, *T* = 240–300 min).

### 2.9. Quantification of Citrate, Succinate, and Malate

For quantification of the anions citrate, succinate, and malate in urine and plasma, capillary electrophoresis (P/ACE*™* MDQ, Beckman Coulter) was used. For this purpose, a fused silica capillary was employed with an effective length of 50 cm, an ID of 75 *µ*m, and an OD of 375 *µ*m. Samples of urine and plasma were diluted with 0.1 M NaOH 1 : 30 and 1 : 2, respectively. Analysis was performed using an anion analysis kit (ABSciex, Fullerton, USA). Per analysis, a volume of 300 nL was applied using pressure injection. The subsequent separation was carried out with a voltage of 30 kV and reverse polarity of the capillary. The detection was performed indirectly by employing a photo diode array at a wavelength of 230 nm.

### 2.10. Statistics

Experiments were performed with six animals per experimental group. Data are expressed as mean values ± SEM. Outliers were removed after box-plot analysis. Comparisons of the urinary concentration of measured anions and their particular excreted urinary amount were performed using regular two-way analysis of variance (ANOVA). *p* values of <0.05 (*∗*), <0.01 (*∗∗*), <0.0001 (*∗∗∗*), and <0.0001 (*∗∗∗∗*), respectively, were considered significant.

## 3. Results

### 3.1. Survival and Enzyme Activities

All animals of the moderate shock groups survived the whole experimental time (*T* = 300 min), independently of the administered resuscitation fluid (not shown). Resuscitation with malate-based crystalloid prolonged the median survival time of severely shocked animals compared to RS (MR7 = 225 min, MR13.6 = 245 min, MR21 = 245 min, and RS = 195 min, not shown).

Regarding parameters of organ injury, severe and moderate hemorrhagic shock partly induced distinct increases in enzyme activities (LDH, CK, ASAT, and ALAT, not shown). In some part, animals resuscitated with MR7, MR13.6, or MR21 showed significantly reduced enzyme activities during the observation period compared to the RS-control group, independent of the administered malate concentration.

### 3.2. Effects of RS, MR7, MR13.6, and MR21 on the Plasma Concentrations of Malate, Citrate, and Succinate in Moderate and Severe HS

**Figure 1:**
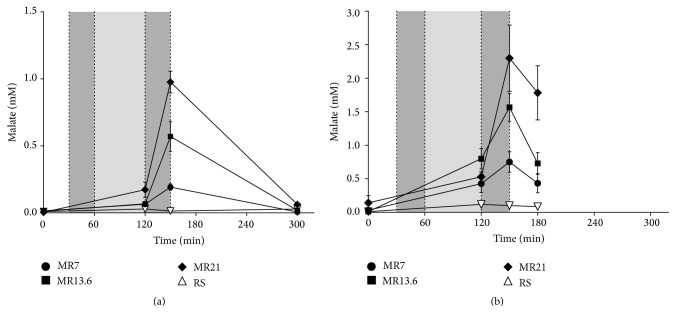
Effects of RS, MR7, MR13.6, and MR21 on the plasma malate concentration in severe and moderate hemorrhagic shock. Rats underwent moderate (a) or severe (b) hemorrhagic shock (dark gray: shock induction; light gray: shock phase; dark gray: resuscitation), were resuscitated with RS, MR7, MR13.6, or MR21, respectively, and were observed until the end of experiment or until the first animal of the RS-control group died. The values plotted are mean ± SEM of 6 individual experiments. Initial and final values were very close to the detection limit (except of initial value in MR21-treated group in severely shocked animals); hence SEM values in these cases are not shown.

Basal plasma malate concentrations were below 0.15 mM (Figures 1(a) and 1(b)). In the moderate hemorrhagic shock group, during the shock induction and shock phase, the plasma malate concentrations only slightly increased but did not exceed initial values (Figure 1(a)). The plasma malate concentrations rose noticeably to 0.19 mM (MR7), 0.57 mM (MR13.6), and 0.98 mM (MR21), respectively, during the resuscitation period depending on the administered malate amount. At the end of the experiment (*T* = 300 min) plasma malate concentrations were returned to initial values of <0.15 mM. In the severe shock group, values rose heterogeneously during the shock induction and shock phase (Figure 1(b)). At the end of the resuscitation phase, malate concentrations increased, depending on the infused malate amount, to 0.75 mM (MR7), 1.57 mM (MR13.6), and 2.30 mM (MR21), respectively. At the end of the experiment (*T* = 180 min, determined by the first animal's death in the RS-control group) malate levels decreased to 0.43 mM (MR7), 0.73 mM (MR13.6), and 1.78 mM (MR21). Values in the RS-control group stayed nearly constant over the whole experimental time.

**Figure 2:**
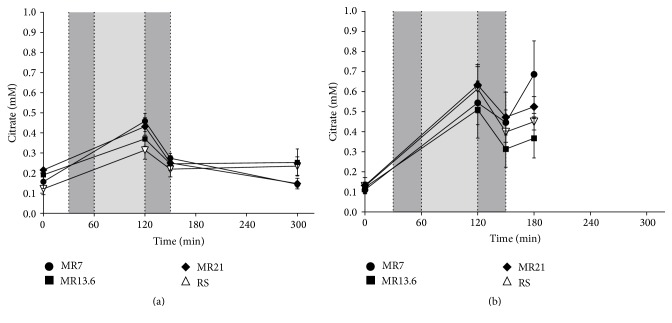
Effects of RS, MR7, MR13.6, and MR21 on the plasma citrate concentration in severe and moderate hemorrhagic shock. Rats underwent moderate (a) or severe (b) hemorrhagic shock (dark gray: shock induction; light gray: shock phase; dark gray: resuscitation), were resuscitated with RS, MR7, MR13.6, or MR21, respectively, and were observed until the end of experiment or until the first animal of the RS-control group died. The values plotted are mean ± SEM of 6 individual experiments. SEM values that are not visible are located behind the symbols.

In the moderate hemorrhagic shock groups basal plasma citrate concentrations were below 0.25 mM (Figure 2(a)). At the end of the shock phase, the concentration rose around the factor 1.5 in all experimental groups. After resuscitation the concentration decreased independently of the administered crystalloid solution and varied between 0.22 mM (RS) and 0.27 mM (MR7). Finally, citrate levels amounted to 0.23 mM (RS), 0.14 mM (MR7), 0.25 mM (MR13.6), and 0.15 mM (MR21). In the severe hemorrhagic shock groups, basal plasma citrate concentrations averaged out below 0.2 mM and slightly increased to about 0.53 mM (MR7, MR13.6) and 0.62 mM (MR21, RS) during the shock induction and shock phase (Figure 2(b)). After resuscitation the concentration decreased independently of the administered crystalloid solution and varied between 0.31 mM (MR13.6) and 0.47 mM (MR21). At the end of the experiment, plasma citrate concentrations averaged out at 0.69 mM (MR7), 0.37 mM (MR13.6), 0.52 mM (MR21), and 0.45 mM (RS).

Succinate plasma concentrations were below 0.1 mM over the whole experimental time, independently of the administered resuscitation fluid (not shown).

### 3.3. Effects of RS, MR7, MR13.6, and MR 21 on the Uroflow in Moderate and Severe HS and the Renal Excretion of Malate, Citrate, and Succinate in Moderate HS

**Figure 3:**
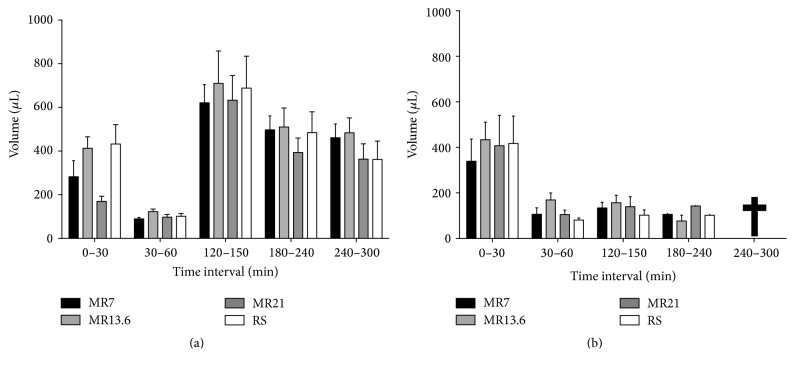
Effects of RS, MR7, MR13.6, and MR21 on the excreted urine volume. Rats underwent moderate (a) or severe (b) hemorrhagic shock, were resuscitated with RS, MR7, MR13.6, or MR21, respectively, and were observed until the end of experiment. Urinary samples were collected immediately after catheter insertion and before shock induction (*T* = 0–30 min) and during shock induction (*T* = 30–60 min), resuscitation (*T* = 120–150 min), and the observation period (*T* = 180–240 min and *T* = 240–300 min). The values plotted are mean ± SEM of 6 individual experiments. SEM values are not shown in the time interval *T* = 180–240 min of severely shocked animals, because animals became anuric or died early, respectively.

The initial urine volume averaged out between 170 *µ*L and 432 *µ*L (Figures 3(a) and 3(b)). During the shock induction phase it clearly decreased, independent of the particular shock depth, to about 110 *µ*L. In the moderate hemorrhagic shock groups, urine excretion clearly increased to 621 *µ*L (MR7), 710 *µ*L (MR13.6), 633 *µ*L (MR21), and 689 *µ*L (RS) during the resuscitation interval (*T* = 120–150 min; Figure 3(a)). In the two subsequent observation intervals (*T* = 180–240 min and *T* = 240–300 min) uroflow slightly decreased, reaching almost initial levels. In contrast, in the severe hemorrhagic shock groups urine excretion stayed nearly constant at shock induction levels during the remaining period of the experimental time (Figure 3(b)).

Renal excretion of malate, citrate, and succinate was only measured under conditions of moderate hemorrhagic shock, due to the small urinary sample volumes in the severe shock groups.

**Figure 4:**
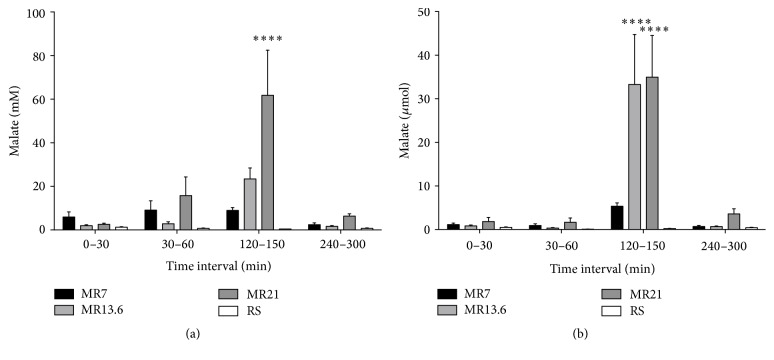
Effects of RS, MR7, MR13.6, and MR21 on the urinary concentrations (a) and absolute amounts (b) of malate. Rats underwent moderate hemorrhagic shock, were resuscitated with RS, MR7, MR13.6, or MR21, respectively, and were observed until the end of experiment. Urinary samples were collected immediately after catheter insertion and before shock induction (*T* = 0–30 min) and during shock induction (*T* = 30–60 min), resuscitation (*T* = 120–150 min), and the last third of the observation period (*T* = 240–300 min). The values plotted are mean ± SEM of 6 individual experiments. ^*∗∗∗∗*^ *p* < 0.0001 compared with the RS-control group.

Urinary malate concentration clearly increased during the resuscitation interval in a dose-dependent manner, being statistically significant in the MR21-group compared to RS-control (Figure 4(a)). Renal malate excretion never exceeded 5 *µ*mol in the initial interval (*T* = 0–30 min) and the shock induction interval (*T* = 30–60 min) (Figure 4(b)). In the resuscitation interval (*T* = 120–150 min) the urinary malate amount clearly rose up to 8 *µ*mol (MR7), 33 *µ*mol (MR13.6), and 35 *µ*mol (MR21), being statistically significant (MR13.6, MR21) compared to RS-control. In the last third of the observation period (*T* = 240–300 min) the excreted malate amount again did not rise above 5 *µ*mol.

**Figure 5:**
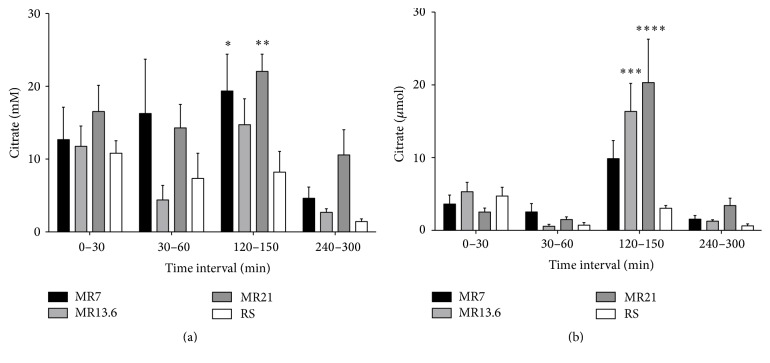
Effects of RS, MR7, MR13.6, and MR21 on the urinary concentrations (a) and absolute amounts (b) of citrate. Rats underwent moderate hemorrhagic shock, were resuscitated with RS, MR7, MR13.6, or MR21, respectively, and were observed until the end of experiment. Urinary samples were collected immediately after catheter insertion and before shock induction (*T* = 0–30 min) and during shock induction (*T* = 30–60 min), resuscitation (*T* = 120–150 min), and the last third of the observation period (*T* = 240–300 min). The values plotted are mean ± SEM of 6 individual experiments. ^*∗*^ *p* < 0.05, ^*∗∗*^ *p* < 0.01, ^*∗∗∗*^ *p* < 0.001, or ^*∗∗∗∗*^ *p* < 0.0001 compared with the RS-control group.

Citrate concentration in the urine significantly increased while animals were resuscitated with MR7 or MR21, respectively, compared to RS-control (Figure 5(a)). Renal citrate excretion never exceeded 7 *µ*mol in the initial interval (*T* = 0–30 min), while in the shock induction interval (*T* = 30–60 min) (Figure 5(b)) excretion decreased in all experimental groups not rising above 3 *µ*mol. In the resuscitation interval (*T* = 120–150 min) the urinary citrate amount clearly increased to 9.9 *µ*mol (MR7), 16.4 *µ*mol (MR13.6), and 20.3 *µ*mol (MR21), being statistically significant (MR13.6, MR21) compared to RS-control. In the last third of the observation period (*T* = 240–300 min) the excreted citrate amount did not rise above 4 *µ*mol.

**Figure 6:**
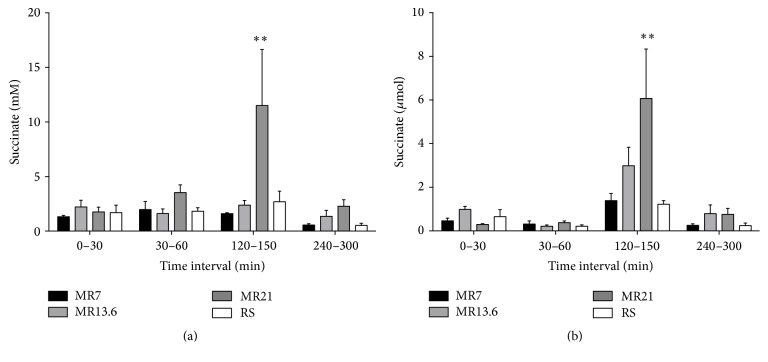
Effects of RS, MR7, MR13.6, and MR21 on the urinary concentrations (a) and absolute amounts (b) of succinate. Rats underwent moderate hemorrhagic shock, were resuscitated with RS, MR7, MR13.6, or MR21, respectively, and were observed until the end of experiment. Urinary samples were collected immediately after catheter insertion and before shock induction (*T* = 0–30 min) and during shock induction (*T* = 30–60 min), resuscitation (*T* = 120–150 min), and the last third of the observation period (*T* = 240–300 min). The values plotted are mean ± SEM of 6 individual experiments. ^*∗∗*^ *p* < 0.01 compared with the RS-control group.

Urinary succinate concentration rose significantly in the experimental groups receiving MR21 during the resuscitation interval (Figure 6(a)). Only in the resuscitation time interval (*T* = 120–150 min) did the renal succinate excretion increase slightly to 1.4 *µ*mol (MR7) and 3 *µ*mol (MR13.6) and increase significantly to 6 *µ*mol (MR21), while in the last interval (*T* = 240–300 min) again initial excretion levels of <1 *µ*mol were reached (Figure 6(b)).

## 4. Discussion

Although malate is a standard component in fluid therapy, information is lacking about its plasma distribution, renal excretion, and metabolism after administration. In the present experiments, malate rapidly disappeared from the blood. Since only a minor amount of the infused malate was eliminated via the kidneys, the vast majority was obviously metabolized.

In moderate HS, plasma malate concentrations sharply increased in a dose-dependent manner at the end of the resuscitation interval but decreased to baseline values within 2.5 hours (Figure 1(a)). Assuming that malate's plasma clearance is nearly linear, a half-life of about 1 hour can be derived. On the other hand, most drugs that are administered intravenously are proportionally eliminated to their plasma concentration (first-order kinetics) [27, 28]. In turn, that means a half-life of 1 hour is overestimated. This is also supported by the results of malate's plasma clearance in severe HS. Even under these conditions (expecting a more decelerated metabolism compared to moderate HS), plasma malate concentrations showed a steep decline, partly up to 54%, already 30 min after resuscitation had ended (Figure 1(b)). These findings suggest that the plasma clearance of malate should be less than 1 hour. Moreover, in moderate HS only 5% of the infused malate was renally excreted, whereupon the vast majority had already been excreted within the reperfusion interval (Figure 4). In severe HS, an increased renal excretion is virtually excluded, since uroflow is excessively impaired (Figure 3(b)). Therefore, based on the present results, it can be concluded that malate's half-life is between 30 min and 60 min under hemorrhagic conditions. These results are in line with the observations by Rietbrock et al. who determined a half-life for malate of 22–24 min in healthy dogs [29]. Furthermore, malate's rapid removal from the blood as well as its excretion profile indicates uptake into the cells with subsequent metabolism (see below).

Apart from the liver [30], skeletal muscle seems to be an important malate consumer, since studies showed malate's positive impact in treating fibromyalgia [31] and its influence on the exercising muscle [6], both probably related to an improved energy metabolism. An ubiquitous metabolism is further supported by the present results. Malate rapidly disappeared from the blood (see above) and the increases in the absolute urinary amounts of the two TCA intermediates citrate and succinate suggest an additional participation of the kidneys in malate's metabolism after infusion. In line, increased renal excretion of citrate following malate has also been reported in healthy test subjects [32–34].

Of the metabolizable anions usually used in fluid therapy, acetate's plasma clearance after infusion follows first-order kinetics, being absorbed within minutes [29, 35]. Acetate has been reported to be renally excreted from less than 10% (in healthy volunteers) [36] up to 40% [37] under the special condition of acetate's application right into the left renal artery in an experimental dog model. Acetate can be metabolized along with the liver by the heart, adipose tissue, kidneys, and muscle [38]. Along with malate and acetate, lactate is a common component in infusion solutions but is metabolized mainly in the liver [39]. This fact presupposes a sufficient liver function, which is typically impaired in terms of hemorrhage and hypovolemic conditions, respectively, leading to adverse effects of the accumulated endogenous lactate and the given lactate during resuscitation (e.g., exacerbated lactic acidosis, inhibition of glycolysis) [17, 18, 40–42]. The adverse effects are additionally fostered by nonexistent renal elimination of lactate [43].

Previous experiments resulted in malate's predominance compared to Ringer's lactate and Ringer's acetate in resuscitation of hemorrhagic shock independent of the shock depth, regarding, for example, survival [15]. In line with these results, regarding malate's protective properties, we could show that MR increased the median survival time after severe hemorrhagic shock, compared to pure RS, independently of the administered malate concentration. The prolonged survival of shocked animals that were resuscitated with MR may be due to a less severe organ injury, which is indicated by the reduced release of intracellular enzymes. In turn, the lower tissue damage could be closely associated with the nearly ubiquitous metabolism of malate, thereby being rapidly available, for example, as precursor for adenosine triphosphate production, for various organs.

## 5. Conclusion

In conclusion, in the present experiments malate showed delayed plasma half-life compared to acetate's literature references. Nevertheless, the still rapid disappearance from the circulation and the marginal renal excretion during the experimental time is of high interest, because the administered malate dosage is virtually completely available to exert its therapeutic actions. Malate's protective effect in the treatment of HS is probably related to its rapid incorporation into the tissues and thereby, for example, being rapidly available as a precursor for adenosine triphosphate production. The nearly ubiquitous metabolism of malate may prevent a potential overload of specific tissues thus giving it a clear advantage over lactate-containing infusion solutions.
